# Socio-cognitive load and social anxiety in an emotional anti-saccade task

**DOI:** 10.1371/journal.pone.0197749

**Published:** 2018-05-24

**Authors:** Mel McKendrick, Stephen H. Butler, Madeleine A. Grealy

**Affiliations:** 1 School of Life Sciences, Heriot-Watt University, Edinburgh, United Kingdom; 2 School of Psychological Sciences and Health, University of Strathclyde, Glasgow, United Kingdom; Brown University, UNITED STATES

## Abstract

The anti-saccade task has been used to measure attentional control related to general anxiety but less so with social anxiety specifically. Previous research has not been conclusive in suggesting that social anxiety may lead to difficulties in inhibiting faces. It is possible that static face paradigms do not convey a sufficient social threat to elicit an inhibitory response in socially anxious individuals. The aim of the current study was twofold. We investigated the effect of social anxiety on performance in an anti-saccade task with neutral or emotional faces preceded either by a social stressor (Experiment 1), or valenced sentence primes designed to increase the social salience of the task (Experiment 2). Our results indicated that latencies were significantly longer for happy than angry faces. Additionally, and surprisingly, high anxious participants made more erroneous anti-saccades to neutral than angry and happy faces, whilst the low anxious groups exhibited a trend in the opposite direction. Results are consistent with a general approach-avoidance response for positive and threatening social information. However increased socio-cognitive load may alter attentional control with high anxious individuals avoiding emotional faces, but finding it more difficult to inhibit ambiguous faces. The effects of social sentence primes on attention appear to be subtle but suggest that the anti-saccade task will only elicit socially relevant responses where the paradigm is more ecologically valid.

## Introduction

Social anxiety can be induced by various social contexts including real or imagined performance situations, personal interactions or being observed in public [1]. Information processing biases such as increased or reduced attention to faces have important implications for the developmental trajectory of social anxiety [2, 3]. Indeed, Bogels and Mansell [4] have highlighted the potential relationship between attentional control (i.e., the way that individuals can control their attention towards a goal-oriented stimuli and ignore an irrelevant stimuli) and attentional biases in social anxiety. However, socially anxious individuals may differ in their ability either to control their selective attention or their ability to ignore irrelevant stimuli such as socially threatening faces. Our ability to attend to task relevant but ignore task-irrelevant stimuli relies on the interaction between bottom up sensory processing (salience driven) and top-down (goal driven) control. Valence (i.e. the degree to which a stimulus is associated with threat or reward) is an important component of salience [5]. Attentional Control Theory [6] models the influence of anxiety on performance and has four central tenets.

Firstly, anxiety impairs the functioning of the attention-like central executive [7]. Following from this, functions of the central executive are differentially vulnerable to anxiety, with influences being detectable on the inhibitory and attentional shifting roles of the central executive (see [8, 9]). Failures to inhibit task irrelevant stimuli and responses can lead to task performance disruption, whilst shifting impairments impede the efficient and flexible allocation of our attention to the current set of task relevant stimuli. The final tenet of the theory relates to the argument that anxiety generally impairs processing efficiency more than performance effectiveness [6, 10].

Automatically generated expectancies of negative outcomes in social situations are believed to be a hallmark of social anxiety. Rapee and Heimberg [11] argue that individuals with social anxiety are overburdened by attention to social cues of a negative nature, whilst Clark and Wells [12] would argue that social anxiety is exacerbated by a reduced attention to positive social cues. To date though the data from studies using static face processing paradigms has provided mixed evidence for either model. Some studies have provided evidence for enhanced attention to threat faces [13, 14, 15, 16, 17] others have found that this is followed by avoidance [14, 15, 18, 19, 20, 21]. Yet other studies have failed to find *any* evidence of attentional biases in socially anxious people [22, 23, 24, 25, 26]. Such conflicting results should of course be viewed in the context of the diverse paradigms, stimuli, sample sizes and social anxiety measures used, and highlight a number of unresolved issues in the field; much is still missing from our understanding of face processing in social cognition.

The interpretation of facial expressions does not in reality occur in isolation from the context in which faces are viewed. This may go some way to accounting for the equivocal findings in the literature. An event such as having to make a speech is an unsettling social engagement for socially anxious individuals because they have tangible social cues to respond to [27]. Such anxiety provoking situations are not captured in a static face paradigm, and studies that have used this technique lack adequate representation of the cues and threats that are encountered in real social situations. In performance situations, if the anxious person expects to have a negative evaluation of their social performance (i.e. indicating that the audience is bored or unimpressed) then seeing a frowning or bored face may elicit enhanced salience from such a face, reinforcing their negative expectations.

However, being thus aware, the individual may also be able to increase their efforts to concentrate on performing well, which may lead to apparent avoidance of the face. Therefore, congruency between the expectation of a social evaluation and the facial expression may have a direct bearing on the individual’s ability to rapidly process and respond to the faces. If the perceived facial expression is not consistent with expectations (i.e. positive or ambiguous), the processes may be slowed whilst the expression is re-evaluated. Since studying information biases in realistic everyday situations is highly challenging, one way to improve the ecological validity of static face paradigms, and their influence on real behavior, would be to increase the socio-cognitive load experienced by the individual during the task, by introducing factors which enhance the self-relevance of the facial expression experienced. This could be achieved either by presenting a social stressor, whereby the participant is aware that the static face task will be followed by a live performance which will be evaluated, or by introducing a social context more directly through priming techniques.

These methods when applied to traditional static face processing tasks may elicit effects that have previously been masked. For example, in a laboratory setting the anti-saccade task [28] has the potential to be an excellent paradigm to assess anxiety related deficits in attentional control. Anti-saccade tasks measure inhibition by requiring participants to ignore an attention grabbing irrelevant stimulus (thereby suppressing a prepotent reflexive response) and make a volitional saccade in the opposite direction. A pro-saccade task on the other hand merely requires the generation of a stimulus driven, reflexive saccade to a target. To perform an anti-saccade, participants are required to suppress the urge to be drawn to a target which would naturally elicit automatic allocation of attention and make a saccade away from it so as to deliberately focus their attention away from the target. Target stimuli in such tasks can be as simple as monochrome shapes or as complex as real-world scenes. Performance is typically measured in terms of rates of erroneous pro-saccades to stimuli and saccadic latencies of successful anti-saccades (e.g. [29]).

Antisaccade tasks have been used in samples with generalised anxiety disorder (GAD). For example, Derakshan et al. [30] found that a high compared to low anxiety group exhibited longer latencies to the first correct saccade when the face was angry relative to happy or neutral faces. This suggests that increased anxiety leads to processing inefficiency to threat because of difficulties inhibiting the threat stimulus but that it does not impact on performance, as there were no differences in error rates. Rather, trait anxious individuals are likely to compensate by making more effort to employ strategies to control performance [31] in line with Attentional Control Theory [6].

To our knowledge Wieser, Pauli and Muhlberger [32] first tested attentional control in social anxiety using an anti-saccade task, although without any contextual element. They found that highly socially anxious participants rated all avatar facial expressions more negatively than low anxious participants. However, eye movement results indicated that whilst high socially anxious participants made significantly more errors than the moderately anxious participants they did not do so in relation to low anxious participants. A group specific compensatory strategy in the high anxious, relative to low anxious participants, should involve faster saccades away from emotional faces with fewer errors compared to neutral face performance. It is possible that these somewhat paradoxical findings reflect individual differences within groups as individual differences in working memory capacity may be responsible for goal-oriented performance deficits, with those lower in working memory finding it more difficult to inhibit distractions [33].

In order to maximally employ the anti-saccade task to model behaviour in individuals high in social anxiety we would argue that a robust attempt is made to minimise potential differences in how participants approach the task. A demonstrably stronger avoidant pattern of saccadic behaviour may emerge when potential task effects in the anti-saccade task (as indicated by the large standard deviations, e.g. [32]) are reduced by increasing the social threat of the task. This can be attained by adding either a social stressor or providing evaluation information prior to stimulus onset. Indeed, behavioural changes have already been demonstrated in some static face paradigms where threat or stress is manipulated to assess biases in attention in social anxiety, with vigilant attention patterns switching to avoidant patterns under such circumstances [34, 35]. If such a manipulation was undertaken in an anti-saccade task then high socially anxious participants may in fact demonstrate lower error rates and faster saccadic latencies away from threatening faces because they have increased stimulus avoidance responses compared to less anxious individuals.

An additional issue that has not been sufficiently addressed in static face paradigms that attempt to stimulate realistic social behaviors in social anxiety relates to the *nature* of the stimuli employed themselves. It may be a significant challenge for socially anxious individuals to inhibit a neutral face during a performance task because of the emotional ambiguity of such a face. To an individual high in social anxiety, a neutral face, rather than portraying no emotion, may be perceived as a mask of emotion, as a ‘poker face’, and may require increased processing, as has been suggested by the increase in right amygdala activity in response to neutral faces found in previous research [36, 37, 38].

The left amygdala is thought to be more specialised for salience driven fear processing, local, high-spatial frequency information and dynamic processing. In contrast, the right amygdala is implicated in emotional evaluation, low-spatial frequency information, global processing and processing of ambiguous stimuli [39, 40, 41]. The right amygdala may be activated through a sub cortical route involving the superior colliculus and pulvinar [39]. Furthermore, Diano et al. [42] reviewed literature on amygdala responses in the absence of direct attention and concluded that the amygdala can be activated without direct awareness through the superior colliculus and pulvinar.

In addition to differential processing of neutral faces, there is also some evidence that socially anxious individuals process emotional words differently than neutral words. For example, Amir and Bomyea [43] found that individuals with social phobia had greater working memory capacity for emotional than neutral words but the pattern was reversed in non-phobic individuals. This was thought to be due to increased anxiety and rumination during the task, which is in line with notion that constraining cognitive resources results in performance deficits for neutral social stimuli, as advocated by Attentional Control Theory [6].

Bearing in mind the aforementioned individual differences in compensatory strategies, if the social threat is high enough, socially anxious individuals may be more likely to try to avoid emotional faces and less likely to look at the face in error. Yet what may be assumed to be avoidance of an emotional face may in fact be a statistical artifact of comparing performance relative to an eroded baseline ability to inhibit neutral faces because of the ambiguity of the meaning of the expression, and the strain that the socio-evaluative processing may then place on the working memory system [43]. Clearly this has powerful implications for the interpretation of data from studies that employ such stimuli.

To adequately assess performance in an anti-saccade task with neutral stimuli a control stimulus such as an inverted face should additionally be employed. Inverted faces have been shown to be less susceptible to configural processing in particular than upright faces [44, 45] in other words to be less likely to be treated as a whole face 'object'. Indeed, investigating the visual ‘pop out’ effect, Brown, Huey and Findlay [46] found that in peripheral vision, upright faces were processed more efficiently than inverted faces. Furthermore, Gilchrist and Proske [47] showed that an upright face was subject to a higher error rate in the anti-saccade task than an inverted face due to its high-level properties making it more likely to attract attention. Inverted faces are thus perceived as a different class of object to faces as we normally process them, and act as a better control for comparing behaviour to faces.

The current study therefore evaluated attentional control with emotional, neutral and inverted faces across social anxiety levels under conditions of increased socio-cognitive load. Thus, strengthening the design adopted by Wieser et al. [32]. Experiment 1 incorporated a design featuring a social stressor via an anticipated social performance [48]. Experiment 2 employed stimuli preceded by prime statements, similar to those used in our previous study of self-referential primes and social perception in a general sample, which influenced perception but not direct attention in respect of emotional categorisation of facial expressions [49]. For the current experiments, it was hypothesised that there would be faster latencies away from angry faces in the anti-saccade task, reflecting anger as a general social threat, but that this effect would be intensified by social anxiety. Furthermore, there should be lower errors to emotional faces relative to neutral upright faces in the high compared to the low anxiety group, with the low anxiety group exhibiting the opposite pattern.

Experiment 1 is in line with Mullins and Duke [50] who found that self-report post-test state anxiety was significantly higher in participants who had performed a face processing task who were told that they would subsequently be asked to deliver a speech to an audience who would evaluate their performance. Therefore, participants in the current study were given the speech task information prior to completing the anti-saccade task in order to increase their social stress whilst completing the anti-saccade task.

Experiment 2, employed either neutral, positive self-referential or negative non-self-referential primes. We followed the premise that socio-cognitive load will facilitate the aforementioned hypotheses in the neutral prime condition, but that these effects will be attenuated by non-socially threatening primes. A pro-saccade task was also added to Experiment 2 and it was predicted that there would be fewer errors made in the pro than anti-saccade task, as is typically reported in tasks employing both pro and anti-saccade task, e.g. [46, 51].

## Method (Experiment 1)

### Participants

170 Participants were initially recruited to complete an online survey but of these only 92 were willing to complete a subsequent experimental stage. One participant was withdrawn because it transpired that she was being treated for depression. Another was withdrawn because she had a diagnosis of Asperger’s syndrome. One more participant was withdrawn because of an equipment failure during the experiment. Therefore, data from 89 participants were analysed. Participants were recruited through the University of Strathclyde’s virtual learning environment; posters displayed around the campus and local businesses; local classified ads and community websites. Participants were initially invited to participate in return for being entered into a draw for a £50 prize, a £5 Amazon voucher or a course credit (for undergraduate students).

Inclusion criteria included English speakers with normal or corrected-to-normal vision, free from visual deficits. Exclusion criteria included a history of substance abuse for the past two years; a current or recent psychiatric disorder or neurological illness. The study was given ethical approval by the University of Strathclyde School of Psychological Sciences and Health Ethics Committee.

Participants were split into high (32), moderate (27) and low (30) social anxiety groups (HSA, MSA and LSA respectively) based the third percentile of scores, i.e. [52, 53] on the online 8-item Brief Fear of Negative Evaluation scale II (BFNE II) [54] which has an internal consistency coefficient alpha of .97.

General social anxiety was measured on the 17-item Social Phobia Inventory (SPIN) [55] which measures five aspects of social phobia including social inadequacy (i.e. fear and avoidance of social situations); self-esteem (i.e. fear and avoidance of criticism); physiological symptoms (i.e. fear of loss of bodily control); social inferiority (i.e. fear and avoidance of authority) and performance anxiety (i.e. fear of public attention). The scale has generally been found to have very good psychometric properties [56] and is correlated with both the Social Phobia Scale to a high degree, and moderately with the Social Interaction Anxiety Scale [57] with coefficients of (.71) and (.60) respectively [48]. Current negative mood and anxiety were measured by administering the self-report 21 item self-report Beck Depression Inventory (BDI-II) [58, 59] and the Beck Anxiety Inventory (BAI) [60] (Beck, Epstein, Brown & steer, 1988). The BDI-II has high reliability, with a coefficient alpha of .93 for undergraduate students and .92 for clinically depressed participants, whilst the BAI has also been demonstrated to have high internal consistency with a coefficient alpha of .91. Additionally, self-focused attention was measured with the Private and Public Body Consciousness Scale (BCS) [61].

#### Participant demographics

The sample was comprised 51 females and 38 male participants. A 2x3 χ^2^ test showed that the gender was equally distributed across the three anxiety groups, χ^2^ (2) = 2.83, *p* = 0.24. Participant’s ages ranged from 17 to 60 and years of education ranged from 12 to 24 years. A series of one-way ANOVAs were conducted for age, education, BCS, BFNE, SPIN, BDI, and BAI scores between anxiety groups. Main effects and Bonferroni post hoc significance values are presented in Table 1.

**Table 1:** Mean, standard deviations and statistical comparisons for participant demographics: Experiment 1.

Participant characteristics								
	HSA M(SD)	MSA M(SD)	LSA M(SD)	F	*p*	HSA LSA	HSA MSA	LSA MSA
BFNE	30.9(3.1)	23.5(1.7)	16.2(3.3)	167.21	< .001	***	***	***
SPIN	28.6(1.7)	20.5(1.9)	11.5(1.8)	23.8	< .001	***	***	**
BCS	33.5(10.1)	31.9(10.5)	30.0(12.1)	0.78	.46			
BDI-II	12.1(1.3)	9.0(1.4)	6.7(1.4)	4.23	.02	*		
BAI	12.5(9.5)	9.5(1.5)	6.2(1.5)	4.69	.02	*		
Age	23(2)	29(2)	27(2)	3.6	.04^▲^			
Education	16.7(2.6)	16.6(3.8)	16.6(2.8)	.02	.98			

***sig at .001

** sig at .01

* sig at .05

^▲^ Bonferroni contrasts were not significant

Participants did not differ in terms of age or education across the three groups. Additionally, no differences between groups were observed for self-focused attention measured with the BCS [61]. However, the three groups were statistically differentiated on measures of anxiety and depression. On the BFNE measure, a Welchs’ test confirmed this. Bonferroni contrasts showed that the HSA group had significantly higher fear of negative evaluation scores than the MSA and LSA groups, with additional differences observed between the latter two groups. A one-way ANOVA on SPIN scores also revealed group differences. Follow-up analysis indicated that HSA participants had significantly higher scores than MSA and LSA groups, with the latter two groups also significantly differing. Additionally, general anxiety scores on the BAI were compared. Following a significant ANOVA main effect contrasts revealed that HSA participants had significantly higher general anxiety scores than LSA participants. However, there were no significant differences between the MSA group and either the HAS or LSA group. Finally, for depression scores, an ANOVA on BDI data was significant, with subsequent Bonferroni contrasts revealing that HSA participants had significantly higher depression scores than LSA participant, but did not differ from MSA participants. Furthermore, there was no significant difference between the MSA and the LSA groups.

### Anti-saccade task

30 photo face stimuli: 5 male (models 21M; 24M; 28M; 33M; 34M) and 5 female models 03F; 05F; 06F; 08F; 09F) displaying angry, happy and neutral expressions were selected from the NimStim Set of Facial Expressions [62]. A control (inverted neutral face) stimulus was also included, from each model. Stimuli were presented twice for a total of 80 trials, and were randomised across participants.

Faces were presented equally to either the right or left of the screen at an eccentricity of 10.1° from the centre of the screen and were presented on a black background at a visual angle of 15.2° in height and 11.3° in width. Dimensions replicated those used by Wieser et al. [32]. Faces were all Caucasian with an even gender balance. Differences in hair and size were controlled by framing the pictures with standardised ovals.

#### Eye movement recording

The stimuli were centrally presented on a 19 inch, 85 Hz Viewsonic G90ft 19 inch colour monitor with its resolution set to 1280 × 1024. Eye movements were recorded with an SR EyeLink II (SR Research, Ontario, Canada) using the centre of the pupil to define pupil position. Eye movements were recorded at a 500 Hz sample rate. Saccade onset was defined as a change in eye position with a minimum velocity of 18°/s or a minimum acceleration of 8000°/s. The programs that controlled the software were designed in-house using Experiment Builder Version 1.10.1025 (SR Research, Ontario, Canada).

### Procedure

The BFNE II [54] was completed online, delivered via Qualtrics survey provider (Qualtrics Ltd, Utah, USA). Consent was obtained by clicking on an onscreen consent box. Participants were then asked to attend the University to complete the experimental phase of the study, prior to which they again read the information sheet and signed a consent form.

During this phase, which lasted for approximately 1 hour, participants were seated 57cm from the monitor and wore a lightweight SR Research headset comprising a head camera and two eye cameras placed just below the eyes. Before commencing the task, participants were asked to complete the Social Phobia Inventory (SPIN); Beck Depression Inventory II [58, 59]; Beck Anxiety Inventory [60] and the Body Consciousness Scale. Questionnaires [61] were administered prior to the task because of the potential effect of the task priming responses.

Following questionnaire completion, participants were told that they would first be asked to participate in a short face processing task and that this would be followed by an interview in front of a live panel who would be evaluating their performance. They were not given any specific information about this. The subsequent anti-saccade task was preceded by 12 practice trials where participants were offered an opportunity to ask for clarity on the task instructions.

Immediately prior to the task, a nine-point calibration sequence was conducted, which was repeated at the start of each trial block. Participants gaze was recorded while fixated on a central white fixation dot with a diameter of 0.5° presented on a black screen and by thereafter tracking their gaze as they followed the dot sequentially around the nine grid points on the screen. Successful calibration was followed by a repetition of the process during the validation stage. At the start of each trial, participants were presented with a central drift correction fixation dot for 500ms, which they were asked to fixate before being presented with a face to either the left or right of the screen for 1000ms.

### Data analysis

Data were analysed using SR Research Data Viewer version 1.9.1 (SR Research Ltd, Ontario, Canada). The first saccade after onset of the face was taken as the saccadic measure. Latencies were calculated from the target onset to the onset of the first saccade and latencies of <80ms were discarded as anticipatory saccades. Initial fixations which were >1° from the central fixation point were also excluded as were saccadic amplitudes under 2°. This led to an average of 4.63% of trials for each participant being excluded. Percentages of erroneous anti-saccades and mean latencies were analysed relative to the number of valid trials across each participant. Eye movement data were analysed in a series of mixed 3 x 4 ANOVAs with one between groups factor: anxiety (high/moderate/low) and one within group factor: face type (angry upright; happy upright; inverted neutral and neutral upright). Dependent variables included mean latency from central fixation point to target for correct anti-saccades and the mean percentage of erroneous pro-saccades.

## Results

### Latency data

A 3x4 repeated measures ANOVA with one between group factor (anxiety) and one repeated measures factor (face type) was conducted on saccadic latency data. Although the data failed the Kolmogorov-Smirnov tests for the HSA group on angry faces, *k-s*(32) = 0.16, *p* = 0.04, and for the MSA group with inverted faces, *k-s*(27) = 0.2, *p* = 0.01, it was normal for all other variables. However, Mauchly’s test indicated that the assumption of sphericity had been violated, (χ^2^ (5) = 677.8, *p*< 0.001) therefore degrees of freedom were corrected using the Greenhouse Geisser estimates of sphericity (ε = 0.69). However, whilst the Levene’s test for each face type were non-significant for angry and inverted faces (p > 0.07), it was significant for happy faces, *F*(2,86) = 4.46, *p* = 0.01 and for neutral faces *F*(2,86) = 5.62, *p* = 0.01.

As ANOVA may not be robust enough under these conditions as the appropriate technique for subsequent analysis [63] the results were analysed using a series of non-parametric tests. A Kruskal-Wallis test revealed no differences between anxiety groups for angry faces, *H*(2) = 0.36, p = 0.83; happy faces, *H*(2) = 0.51, p = 0.77; inverted faces, *H*(2) = 4.57, p = 0.1 or neutral faces, *H*(2) = 0.69, *p* = 0.71. Additionally, a Friedman’s test was conducted to examine potential differences in response latencies across emotions. This analysis revealed a borderline effect, χ^2^(3) = 7.83, *p* = .05. Follow-up Bonferroni-corrected Wilcoxon Signed Ranks tests for 6 comparisons (setting an alpha of .0083) revealed lower latencies to respond to inverted faces (*Mdn* = 255.79 ms) relative to happy faces (*Mdn* = 263.56 ms) *Z* = 2.86, *p* = .004, *r* = .30. There was also a directionally similar borderline significant difference between inverted faces and angry faces (*Mdn* = 261.68 ms), *Z* = 2.50, *p* = .001, *r* = .30. The remaining four comparisons inverted v neutral; happy v neutral; angry v neutral and happy v angry were all non-significant (p = .15; p = .16; p = .11; p = .73) respectively.

### Error data

A 3x4 repeated measures ANOVA was conducted, with one between group factor (anxiety) and one repeated measures factor (face valence) for error rate. Although the data failed the Kolmogorov-Smirnov tests primarily due to a skew towards 0 scores, Mauchly’s test indicated that the assumption of sphericity could be assumed, (χ2(5) = 7.8, *p* = 0.17) and the Levene’s test for each face type were non-significant (p> 0.43) so ANOVA should be robust enough under these conditions to use for the analysis [62]. Results revealed no significant main effect for face valence, *F*(3,258) = 0.67, *MSE* = 0.05, *p* = 0.57, *Pη*^*2*^ = 0.01, *power* = 0.19 and no significant main effect of anxiety, *F*(2, 86) = 77.98, *MSE* = 0.09, *p* = 0.61, *Pη*^*2*^ = 0.01, *power* = 0.13 or anxiety x valence interaction, *F*(2, 86) = 2.33, *MSE* = 0.01, *p* = 0.1, *Pη*^*2*^ = 0.05, *power* = 0.46.

## Discussion

Participants were asked to perform an anti-saccade task to emotional, neutral and inverted faces whilst under a condition of apprehensive social stress. It was predicted that the social stressor would induce faster latencies away from angry faces in the anti-saccade task, and that this would be intensified by degree of social anxiety. However, no differences in latencies were found across anxiety groups or face types. There was some evidence however of a face inversion effect with a significant difference between upright happy and inverted faces and a borderline effect for the difference in latencies between angry upright and inverted faces.

This latter finding only partially supports previous findings such as Brown, Huey and Findlay [46] which suggested that upright faces were processed more efficiently, since no inversion effect was found for neutral upright compared to inverted faces. It is possible that the social relevance of inverted neutral faces may have been inflated by cognitions and anxiety about the anticipated concept of being evaluated, regardless of participants’ dispositional social anxiety.The effect of increased social salience may have been stronger for error rates which showed no evidence of inversion effects.

Furthermore, it was hypothesised that there would be lower errors to emotional faces relative to neutral upright faces in the high compared to the low anxiety group, who would exhibit the opposite pattern. Again, this hypothesis was not supported as no significant group differences or effects of face type were found. These findings differ from those of Wieser et al. [32] who found differences in error rates across the high and moderate anxiety groups.

It is possible that the social stressor was not strong or consistent enough to demonstrate the predicted effect. By providing a greater controlled context in experiment 2, we may be able to homogenise the degree of social attachment that participants feel to the stimuli they are exposed to, and more readily control the degree of socially influenced attention that participants apply to experimental trials. In the first study a speech threat was used but it is possible that although there may have been some low-level awareness of this, the mechanical task demands may have remained powerful enough to distract subsets of participants enough to forget the forthcoming speech task for periods during task engagement. Therefore, the anticipated effects that would be driven by the forthcoming speech task may have again been masked to some extent. Experiment 2 should provide a stronger test of our thesis.

## Experiment 2

Socially anxious individuals are thought to draw upon negative self-relevant memories, which in turn lead to expectations of negative evaluation in social situations. Thus as Rapee and Heimberg [11] suggested, attention to social information is dependent on congruency between expectations and perception. Increasing the social evaluative context of static emotional faces may elicit the types of responses that would more naturally occur in a social situation than would occur in typical static face paradigms. A highly socially anxious person is likely to generate a continuous series of negative self-referential thoughts throughout a task rather than to generate these prior to the task [11] as was the case in Experiment 1. Increasing socio-cognitive load by introducing contextual factors through priming may result, in itself, in a constantly updated evaluation-based attention cycle.

Indeed, we have successfully demonstrated the influence of sentence primes on cognitions, particularly when primes and emotional expressions have been congruent. Our findings indicated that in a general population, negative self-referential primes reduced self-esteem, particularly when paired with a face perceived as negative [49]. We anticipated that socially anxious individuals would automatically generate such self-referential negative primes. Therefore, we did not seek to add an additional layer of threat to the current study, but to enhance the self-relevance of the task to make it more ecologically valid.

When introducing primes with a social component care must be taken to ensure the manipulation is merely adding a socio-cognitive load. The purpose of such primes should be to afford a measure of the effect of generic socio-cognitive load rather than impending social threat. Therefore, the social prime types selected should be non-threating in nature. One way of achieving this is to direct negative social information away from the participant and towards another hypothetical character. Another is to direct positive social information towards the participant. In addition to neutral primes, we shall employ both types of non-threatening social prime in the following experiment.

It should be stated that we do not view neutral primes to be merely ‘filler’ stimuli in this study, rather we anticipate that they shall effectively foster natural levels of background thought in participants, which in the case of individuals with higher levels of social anxiety should be very likely to feature the generation of negative self-referential thoughts. Conversely, non-self-referential primes and positive self-referential primes may reduce the personal social threat of the faces viewed, compared to the natural generation of a negative self-referential internal narrative that socially anxious individuals would be expected to generate in social contexts, and in the context of our neutral primes. A positive self-referential prime should lead a participant to expect that a non-socially threatening face would appear afterwards in the context of an anti-saccade task. Similarly, a negative non-socially threatening prime, whilst negative, should not signal a social threat because it directs the negative response away from the participant to another cause. This means that what is being investigated is simply the effect of socio-cognitive load, where increased socio-evaluative processing may occur, rather than a threat prime where threat specific processing is likely to occur.

It was expected that socially anxious individuals would be likely to generate negative self-referential thoughts in response to a neutral prime where a direct reason for the expression of the face was not provided. In contrast, non-socially threatening emotional primes that did explain the reason for the expression would be expected to attenuate the generic effect of socio-cognitive load in a way that neutral primes are unlikely to do. Thus, in this experiment wel had a prime type that mimics reality by providing non-directive thoughts to accompany the visual stimuli (with their inherent capacity to facilitate participants thought processes), and two prime types that had a clear social component which enhance the social salience of the task without increasing social threat.

We added a pro-saccade task for Experiment 2 to ensure task effectiveness since we were introducing an additional variable, thereby making the task more complex. We expected to observe fewer errors made in the pro-saccade than in the anti-saccade task, and lower latencies for correct saccades in the pro-saccade condition relative to the anti-saccade condition. With specific regard to the primes employed in experiment 2, Van Peer et al. [64] have suggested that there is a general approach-avoidance response for happy and angry faces. This may reflect the strength of angry faces in terms of negative evaluation whilst happy faces indicate positive evaluation. It was therefore hypothesised that there would be a general approach-avoidance response for happy and angry faces, characterised by longer latencies to saccade away from happy faces and shorter latencies for angry faces, but that this effect would be intensified by the groups' degree of social anxiety, particularly with neutral primes. Furthermore, there would be lower errors to emotional faces relative to neutral upright faces in the high compared to the low anxiety group in the anti-saccade task with neutral primes, but this latter effect would be attenuated by non-socially threating primes.

## Method

### Participants

Initially, 136 participants were recruited through adverts displayed around the campus and local businesses; local classified ads and community websites. Participants were invited to participate in return for being entered into a draw for a £50 prize. Inclusion and exclusion criteria were as Experiment 1. All participants provided informed consent to take part in the study, and local ethical approval was again obtained.

From the original survey respondents 76 participants who were naive to the purpose of the study completed the experiment. However, two participants were withdrawn as they had clinical levels of depression and revealed after the experiment that they were receiving medication to treat this. Another two participants were withdrawn as their level of English was deemed not to be at a level to have understood the task instructions. Thus, data from 72 participants were analysed. Participants were again split into high (24), moderate (24) and low (24) anxiety groups (HSA, MSA and LSA respectively) based the third percentile of scores on the online 8-item BFNE II [54].

Social anxiety was again also measured using the SPIN, whilst current negative mood and anxiety were measured by BDI-II and BAI respectively. Additionally in this study, to investigate the relationship between self-report attentional control and performance, participants completed the 20-item Attentional Control Scale (ACS) [65], which has a high internal consistency with a coefficient alpha of .88. The ACS is a self-report questionnaire which measures focused attention including inhibition and task shifting, with items such as “When I need to concentrate and solve a problem, I have trouble focusing my attention” and “It is easy for me to alternate between two different tasks”. Responses on a four point Likert scale range from, 1 (almost never) to 4 (always). Scores can range from 20 to 80 with higher scores indicating stronger attentional control.

### Participant demographics

The sample was comprised of 51 female and 21 male participants but a 2x3 χ^2^ test showed that the gender was equally distributed across the three groups, χ^2^ (2) = 2.82, *p* = .24. Participant’s ages ranged from 18 to 57 and years of education ranged from 11 to 24. A series of one-way ANOVAs were conducted for age, education, BFNE, SPIN, BDI, BAI and ACS scores. Main effects and Bonferroni post hoc significance values are presented in Table 2.

**Table 2:** Mean, standard deviations and statistical comparisons for participant demographics: Experiment 2.

Participant characteristics								
	HSA M(SD)	MSA M(SD)	LSA M(SD)	F	*p*	HSA LSA	HSA MSA	LSA MSA
BFNE	31.5(2.9)	22(2.4)	14.6(2.6)	242.74	< .001	***	***	***
SPIN	31(12.9)	20.6(8.6)	12(7.2)	22.56	< .001	***	***	**
ACS	48.4(8.7)	51.6(6.1)	53.7(7.3)	3.07	.05	*		
BDI-II	11.8(7.8)	10.1(6.6)	6.7(6)	3.49	.04	*		
BAI	13(8.2)	10.6(8.1)	6.4(7.2)	.31	.02	*		
Age	26(8)	31(12)	31(11)	2.7	.08			
Education	16.3(2.7)	16.9(3)	16.4(3)	.1	.65			

***sig at .001

** sig at .01

* sig at .05

Participants across groups also did not differ in terms of age or education across the three groups, but were reliably differentiated on social anxiety measures. However, higher scores for the high anxiety group relative to the low anxiety group for both depression and general anxiety were also observed. Additionally, the borderline significantly lower ACS scores for the high relative to the low anxiety group may suggest that the highly socially anxious participants had less confidence in their ability to control their attention than their less anxious counterparts.

### Design

In order to measure the effect of the task, eye movement data were initially analysed in a pair of mixed four way ANOVAs with one between groups factor: anxiety group (high/medium/low) and three within group factors: condition (pro-saccade/anti-saccade); prime (neutral; positive self-referential (SR); negative non-self-referential (NSR)) and face type (angry; neutral; happy; inverted neutral face). The dependent variables were again mean latencies towards or away from the target (in pro and anti-saccade trials respectively), and percentages of erroneous anti-saccades, analysed relative to the number of valid trials across each participant. To simplify the analysis following the investigation of a task effect, a series of mixed three way ANOVAs were then computed for the pro-saccade and anti-saccade tasks separately.

#### Prime validation

In order to validate the neutrality of the neutral primes employed and the effectiveness of the target negative non-self-referential primes as being less threatening than negative self-referential primes, and the positive self-referential primes as being more positively perceived than a positive non-self-referential context, a total of 400 primes were initially generated for the validation procedure. These included 80 primes of each of five sentence types: positive self-referential (SR); negative self-referential (SR); positive non-self-referential (NSR); negative non-self-referential (NSR) and neutral. Each statement was comprised of six words to standardize the length. Seventeen participants were asked to rate both the valence of statements presented on paper on an 11-point scale ranging from –5 (*very negative*) to 0 (*neutral*) to +5 (*very positive*) (after e.g. [66]), and degree of self-reference ranging from –5 (definitely self-referential) to 0 (neutral) to +5 (definitely other-referential).

A repeated measures one-way ANOVA with five levels of valence (positive SR; negative SR; positive NSR; negative NSR and neutral) revealed a significant main effect, *F*(1.14,18.25) = 363.14, *MSE* = 1.15, *p*< .001, *partial η*^*2*^ = .96. Bonferroni comparisons revealed that negative NSR primes (M = -2.37, SE = .01) were rated significantly more negatively than positive NSR primes (M = 2.36, SE = .13, p< .01) or neutral primes (M = 0.02, SE = .01, *p*< .01) but significantly less negative than negative SR primes (M = -2.96, SE = .15). Positive SR primes were rated significantly more positively than negative SR primes (M = -2.37, SE = .13, *p*< .001) and neutral primes (*p*< .001).

Final statements were selected from the original list on the basis of the most extreme valence for emotional primes (i.e. 5 on the Likert scale rather than 4) and the lowest valance for neutral primes (i.e. the primes with ratings closest to 0). A further 1-way repeated measures ANOVA was conducted for the final prime set, which revealed a significant main effect of valence, *F*(1.11, 17.73) = 482.25, *MSE* = .6, *p* < .001, *partial η*^*2*^ = .97. Bonferroni contrasts showed that negative NSR sentences were rated significantly more negatively (M = -3.12, SE = .15) than neutral (M = .01, SE = .01, *p*< .001) or positive SR sentences (M = 3.04, SE = .14, *p* < .001), which were rated significantly more positively than neutral sentences. Although negative NSR primes were rated as more negative than neutral primes, a significant main effect of reference was also found in a 1-way repeated measures ANOVA, *F*(1.23, 19.69) = 319.93, *MSE* = 1.13, p< .001, *partial η*^*2*^ = .95. Bonferroni contrasts showed that negative NRS sentences were rated significantly more as referring to others (M = -3.3, SE = .22) than neutral (mean = 0, SE = 0) or positive SR sentences (M = 3.92, SE = .23), which were rated significantly more self-referential than neutral sentences.

In the interests of reducing the length of the experimental block, which at 400 trials would be extremely long for an eye tracking study in terms of participant fatigue, the number of primes was reduced. It was important to retain the neutral primes to control the narrative to some extent. However, it was reasoned that socially anxious individuals would be likely to generate their own negative self-referential internal narrative, negating the need to provide these additionally. Therefore, by way of comparison, we attempted to reduce the social threat by providing a low threat-controlled narrative through non-threat sentence primes. As the analysis above suggests negative non-self-referential primes are likely to be less socially threatening than neutral primes because they direct the negative information away from the individual. Additionally, we included positive primes directed towards the individual. Thus, in the present study, three prime types were included: neutral, positive (SR) self-referential and negative non-self-referential (NSR). This gave rise to 240 primes (80 neutral, 80 negative non-self-referential and 80 positive self-referential). An example of a positive SR prime was ‘Sara is impressed by your speech’ while a negative NSR prime was, ‘Sara is annoyed with her boss’ and a neutral prime example was ‘Sara takes the train to work’.

### Apparatus and stimuli

Forty face photos were used. This was comprised of 5 male and 5 female models consisting of angry, happy and neutral expressions again selected from the NimStim Set of Facial Expressions [62]. A control inverted neutral face stimulus was also included (only a neutral face was included to keep trial numbers manageable).

Trials (n = 240) were split into two blocks of 120 pro-saccade and 120 anti-saccade trials with 40 trials for each prime type employing the same face stimuli equally in both blocks of trials. Presentation order of blocks was counterbalanced amongst participants. Stimuli presentation order was also randomised across participants. Faces with each prime type were equally presented to the right or left of the screen at a visual angle of 10.1° from the centre, on a black background at a visual angle of 15.2° in height and 11.3° in width. Dimensions again replicated those used by Wieser et al. [32]. Face configurations were again presented on a ViewSonic G90ft 19 inch colour monitor attached to a Phillips personal computer with eye movements again recorded via an SR Research Eyelink 2 (SR Research Ltd, Ontario, Canada) using previously described settings. The programs that controlled the software were designed in-house.

## Procedure

Prior to commencing the eye-tracking task, participants were asked to read the participant information sheet and give consent. Before commencing the experimental task, participants were asked to complete the BFNE II [54], the Social Phobia Inventory (SPIN) [55], the Attentional Control Scale [65], the Beck Depression Inventory II [58, 59] and the Beck Anxiety Inventory [60].

**Fig 1:**
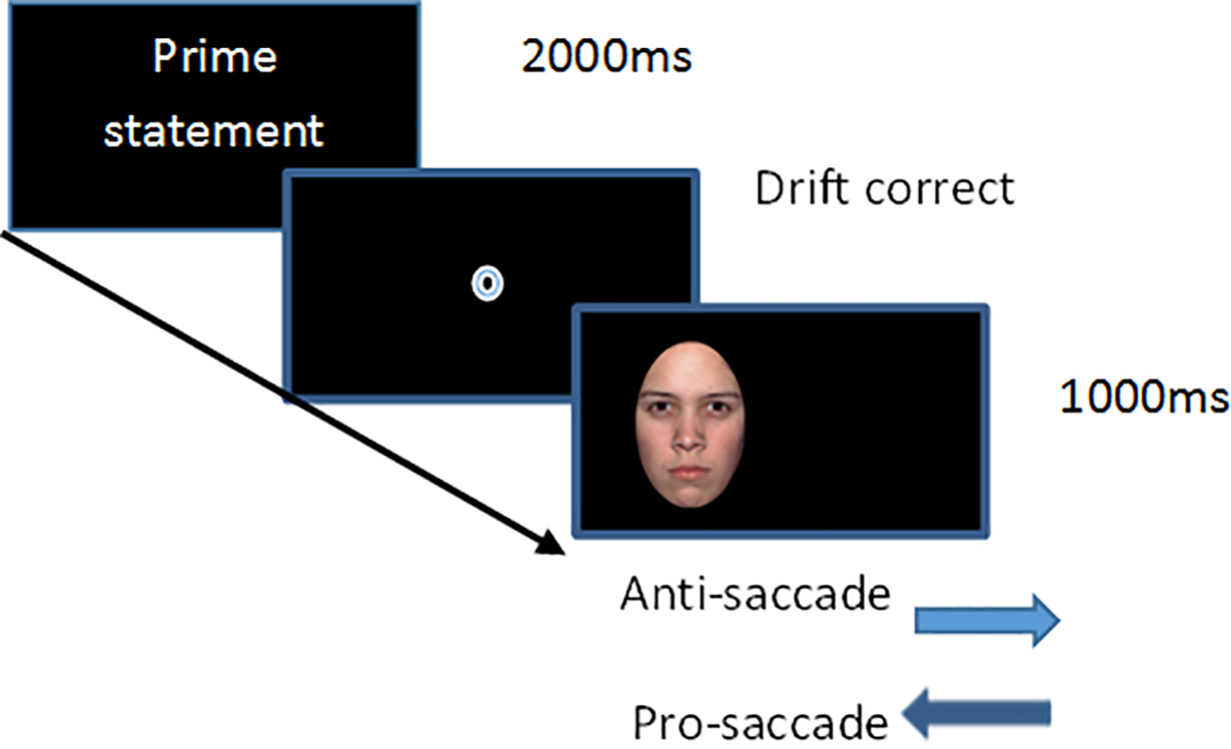
Schematic of modified emotional face saccade task.

Participants were then seated 57cm from the monitor. The experimental task was preceded by 12 practice trials where participants were offered an opportunity to ask for clarity on the task instructions and indicate that they had time to process and understand the meaning of the sentences. Immediately prior to the experimental task, and prior to each trial block participants completed a nine-point calibration and validation sequence. At the start of each trial, participants were presented with a prime statement for 2000ms that they were asked to read. They were then presented with a drift correction where they were asked to fixate a dot before being presented with a face to either the left or right of the screen for 1000ms. On the pro-saccade block they were asked to look towards the face and in the anti-saccade block they were asked to look to the opposite side of the screen to where the face appeared. The task is illustrated in Fig 1.

### Eye movement data preparation

Data were again analysed using SR Research Ltd. Data Viewer 1.9.1 (Ontario, Canada). The first saccade after onset of the face was taken as a saccadic measure. Anticipatory saccades, improper fixations and low amplitude saccades were again excluded, as in experiment 1, resulting in an average of 2.9% of trials for each participants being excluded. Percentages of anti-saccades and pro-saccades were analysed relative to the number of valid trials across each participant.

## Results

As recommended by Field [67] where Mauchly’s test was significant at below .75, the Greenhouse Geisser correction was applied, and where it was significant with a value of above .75, the Huynh-Feldt epsilon was instead applied.

### Latencies

A 2x3x4 ANOVA with three repeated measures factors: task (anti/ pro); prime type (negative/ neutral/ positive) and face type (angry/ happy/ inverted/ neutral) revealed a significant main effect of task for saccadic reaction time, *F*(1,71) = 410.23, *MSE* = 360.32, *p*< .001, *partial η*^*2*^ = .85. Latencies were significantly shorter for pro-saccades (M = 181.36ms, SE = 2.2) than for anti-saccades (M = 265.49ms, SE = 5.05). Thus, the hypothesis that latencies would be shorter in the pro than anti-saccade task was supported.

**Fig 2:**
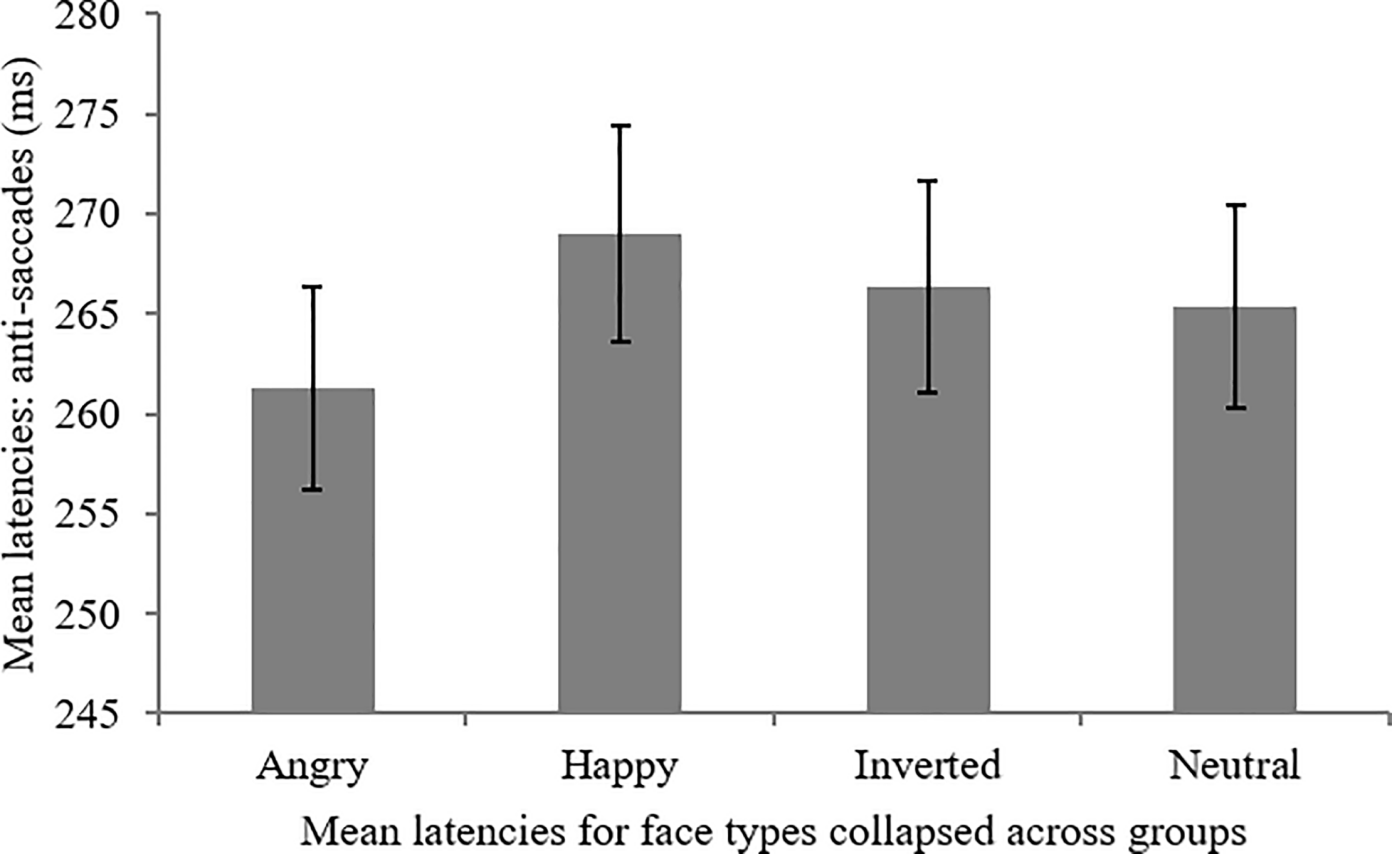
Mean saccadic latency for faces in the anti-saccade task (bars denote standard errors).

An anxiety group (3) x prime (3) x face type (4) ANOVA for the anti-saccade task revealed a significant main effect of face, *F*(3,207) = 4.09, *MSE* = 539.61, *p* = .01, *partial η*^*2*^ = .06. Bonferroni corrected post-hoc comparisons revealed that there were significantly shorter mean latencies before saccading away from angry (M = 261.27ms, SE = 5.08) compared to happy faces (M = 268.98ms, SE = 5.42), *p* < .01. The means and standard errors for saccadic latency for faces are illustrated in Fig 2.

There was no significant main effect of anxiety group on saccade latency for anti-saccades, *F*(2,69) = .42, *MSE* = 22047.53, *p* = .66, *partial η*^*2*^ = .01, *Power* = .12. There was no significant main effect of prime, *F*(1.92, 132.72) = 0.03, *MSE* = 546.63, *p* = .97. There was also no significant anxiety group x prime interaction, *F*(3.85, 132.78) = 1.58, *MSE* = 545.47, *p* = .19, *partial η*^*2*^ = .04, or anxiety group x face interaction, *F*(6, 207) = 0.65, *MSE* = 539.59, *p* = .69, *partial η*^*2*^ = .02. The prime x face interaction *F*(3.87,267.09) = 1.94, *MSE* = 1363.96, *p* = .12, *partial η*^*2*^ = .03 was also not significant. Crucially, the anxiety group x prime x face interaction was also non-significant, *F*(7.74, 267.07) = 1.52, *MSE* = 1364.8, *p* = .15, *partial η*^*2*^ = .04. Therefore, the hypothesis that angry upright faces would overall be associated with faster latencies in the anti-saccade task when compared to other face types was partially supported for happy faces. However, the prediction that this effect would be exaggerated by social anxiety, but attenuated by a non-threat prime, was not supported since there were no significant effects of anxiety group or prime type.

### Accuracy

A three-way repeated measures ANOVA with task (pro/anti); prime (negative/ neutral/ positive) and valence (angry/ happy/ inverted/ neutral) found that the main effect of task for error rates was significant *F*(1,71) = 86.13, *MSE* = .01, *p*< .001, *partial η*^*2*^ = .55. Bonferroni pairwise contrasts revealed that error rates were significantly higher for anti-saccades (M = 17%, SE = .01) than for pro-saccades (M = .01%, SE = .01). Thus, the hypothesis that error rates for correct pro-saccades would be lower than those observed for correct anti-saccades was supported.

A 3 x 3 x 4 (anxiety x prime x face) ANOVA for errors on the anti-saccade task failed to reveal a significant main effect for anxiety group *F*(2,69) = .84, *MSE* = .02, *p* = .44, *partial η*^*2*^ = .02. The main effect of prime was not significant *F*(2, 138) = 2.3, *MSE* = .02, *p* = .74, *partial η*^*2*^ = .04. Nor was the main effect of face type, *F*(2.89, 5.78) = .97, *MSE* = .02, *p* = .41, *partial η*^*2*^ = .09. The anxiety x prime x face interaction was also non-significant, *F*(12, 144) = 1.12, *MSE* = .01, *p* = 0.07, *partial η*^*2*^ = .34, as was the potential interaction for anxiety x prime *F*(4,38) = .45, *MSE* = .01, *p* = .77, *partial η*^*2*^ = .01.

**Fig 3:**
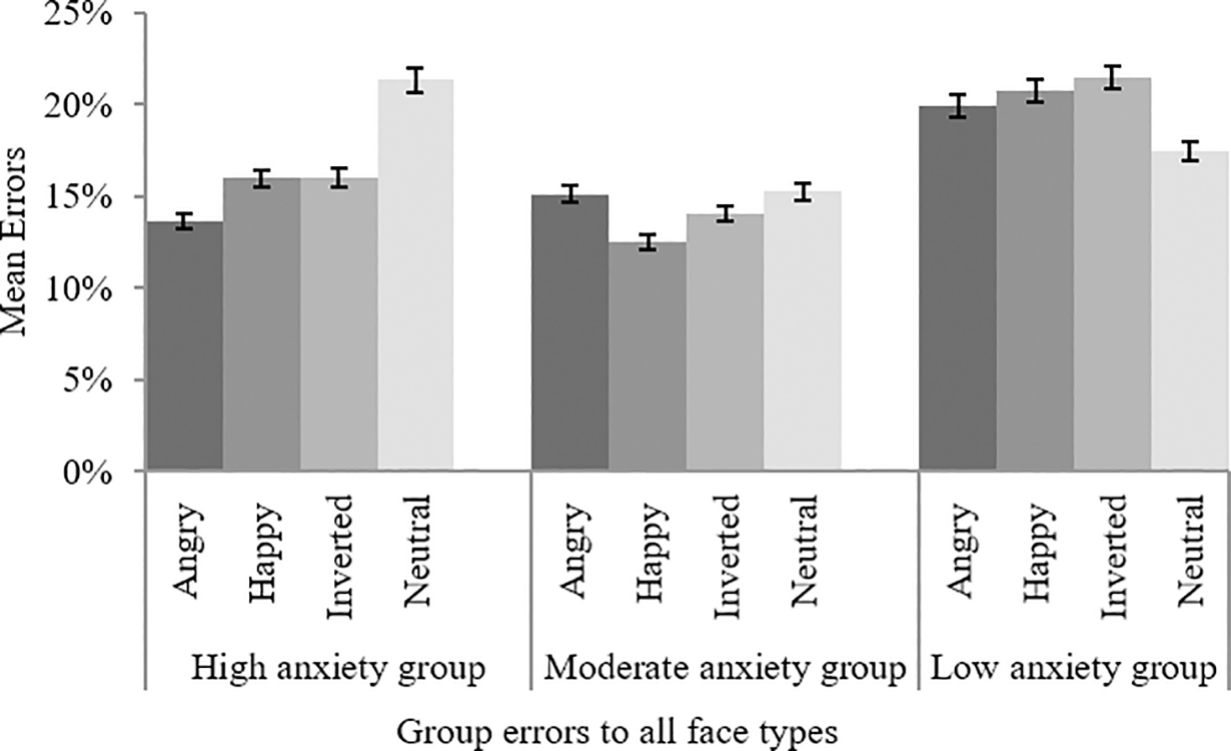
Mean anti-saccade errors across anxiety groups and face types (bars denote standard errors).

Although there were no group differences as predicted, there was a significant anxiety x face interaction, *F*(3.35 199.44) = 3.35, *MSE* = .05, *p* = .004, *partial η*^*2*^ = .09. Bonferroni corrected post-hoc comparisons revealed that error rates were significantly higher in the high anxiety group only for neutral faces (M = 21.4%, SE = 3.3) relative to angry faces (M = 13.6%, SE = 3.3, *p* = .002) and happy faces (M = 16.4%, SE = 3.2, *p* = .03). Thus, in partial support for the hypothesis, regardless of context, the high anxiety group made significantly more errors to neutral upright faces compared to angry or happy faces. The opposite (though non-significant) pattern was observed in the low anxious group. The differences across groups and facial expressions are illustrated in Fig 3.

Finally, because depression and general anxiety scores had been significantly higher in the high anxiety group than the low anxiety group, correlations were conducted for depression and generalised anxiety, with errors across each face emotion and prime type. No significant correlations were found between depression or general trait anxiety and error rates for any of the facial expressions.

## Discussion

Saccadic latencies were as predicted shorter, and fewer errors were made in the pro-saccade than anti-saccade task. There were also significantly shorter latencies before saccades were made away from angry faces compared to happy faces in the anti-saccade task, but there was no significant difference in latencies between angry and neutral faces. This suggests that angry faces were processed efficiently and would indicate an automatic tendency to orient away from them given that an angry face may indicate a social threat. Conversely with happy faces there may be less motivation to look away as it would be associated with reward. This may indicate general approach-avoidance for happy and angry faces respectively as Van Peer et al. [64] have suggested.

Whilst there were no overall group differences for latencies or error data, in the anti-saccade task, the high anxiety group made significantly more errors to neutral upright faces compared to angry and happy faces, whilst the opposite (albeit non-significant) pattern was observed in the low anxious group. This provides some evidence of avoidance of emotional faces in high anxious participants, but conflicts with the findings of Wieser et al. [32] who found that high anxious relative to moderately anxious participants had a higher error rate for all faces.

One explanation for these results is that the high social anxiety group may have had more indecision around neutral faces making it more difficult to inhibit them. This may have been due to the emotional ambiguity of the face. The moderate group had similar error rates for all face types, whilst the low social anxiety group demonstrated the opposite pattern to the high social anxiety group, suggesting that both of the more extreme groups may respond differently to emotional faces relative to neutral faces, but for different reasons. For example, the high social anxiety group may be more sensitive to neutral faces because of the increased socio-evaluative processing required for more ambiguous faces, as we have proposed.

The higher error rate for neutral faces in the high social anxiety group may have been consistent to some extent with the assertion of Carver and Scheier [68] regarding the effect of cognitive load and anxiety in placing strain on the working memory system and precipitating goal disengagement. The results also support Amir and Bomyea [43] who found that individuals with social phobia had greater working memory performance for emotional than neutral words, but that the reverse pattern was observed in non-phobic individuals. This is consistent with the results in the current study since the opposite error pattern was also found in low socially anxious participants, albeit without significant differences. This may suggest that the high social anxiety group engaged in increased evaluative processing of neutral faces, which may represent more ambiguous social cues.

The lower errors made with emotional faces may have been due to avoidance of them relative to neutral faces. This may have been due to the employment of more realistic faces than those used in the Wieser et al. [32] study in which high socially anxious participants had a higher mean error rate to all faces compared to moderately anxious participants. Indeed the error rate for neutral faces in the current study was similar to the error rate for all faces in the high anxiety group observed in the Wieser et al. [32] study.

This explanation is consistent with the findings of Helfinstein et al. [35] that under neutral conditions a vigilant pattern towards threatening faces may emerge with a socially anxious sample, but when the social threat is increased the pattern shifts towards avoidance. Therefore, whilst there were more errors in the Wieser study [32], in the current study participants may have been motivated to avoid emotional faces which they may be able to quickly assess as being socially threatening but experienced more difficulty in interpreting the neutral faces. Additionally, the stimuli used in the current study may have been perceived as being more socially threatening than the avatars used in the Wieser et al. study [32] because (i) they were more realistic looking photographic images of facial emotion and (ii) because they were preceded by socio-evaluative primes which may have inflated the social relevance of the face. This may have led to a pattern that is more consistent with avoidance rather than vigilance to emotional stimuli, indicated by the reduced error rate in the high anxiety group compared to neutral faces.

Individual differences across groups in levels of attentional control make it difficult to interpret the results of anti-saccade tasks to some extent, and the sample must also be considered as a potential reason for the differences between the results of the current study and those of Wieser et al. [32]. However, even taking this into account, there appear to be some similar patterns across these two sets of results. As well as the similarity between the error rates for neutral faces in the current study and all face types in the Wieser et al. study [32] (which did not employ a socio-cognitive load), the pattern for the latency data was analogous. Wieser et al. [32] had a similar, although non-significant pattern of angry faces having shorter saccadic latencies in the anti-saccade task relative to happy faces. The current results may have been more extreme because the faces are preceded by social information which may enhance the salience of the social threat of an angry face resulting in an avoidant response.

Finally, we observed no evidence of an inversion effect in experiment 2. The lack of an inversion effect reinforces the weak effect found in Experiment 1, since the enhanced social relevance of the faces in this experiment facilitated by increased socio cognitive load would be likely to further ameliorate any face inversion effect. We would argue that the primes which lead the viewer in some trials to consider the evaluative meaning of the expression would be likely to cause them to attach more social significance to an upside-down face than they may otherwise do.

## General discussion

The anti-saccade task has superb potential to accurately model the behaviour of individuals high in social anxiety in social cognition research. We have argued that the social context of the task should be strengthened and have proposed that this can be achieved by either introducing a social stressor by way of priming, or by providing forthcoming evaluation information, which we predicted would lead to more replicable group behaviour with regard to reactions to facial stimuli.

In experiment 1, participants were placed under a condition of social stress through the provision of information that they would be required to undertake a speech task after they participated in an anti-saccade task to emotional, neutral and inverted faces. Whilst some evidence of an inversion effect was observed, no group differences in error rates or latencies were seen. Whilst acknowledging these null results may simply indicate no observable effect of social anxiety, it is possible that the social stressor employed may not have been strong enough to pierce through effects driven by individual differences in performance.

Experiment 2 again featured an anti-saccade task. However, it was anticipated that the use of a prime prior to each trial would, through manipulation of three prime types, afford a stronger effect of socio-evaluative stress at regular but unpredictable intervals, thus magnifying the social relevance of the faces employed in the task. Evidence supported this prediction, particularly with regard to the high socially anxious group. Overall, we observed lower saccadic latencies for anti-saccades from angry faces relative to happy faces, which we suggested was indicative of the stronger social threat of such faces, relative to faces we associate with reward.

Even in the absence of full awareness of facial expressions, cognitions can be influenced by threat faces. For example, greater amygdala responses to fear faces have been associated with a negative interpretation bias in the absence of awareness of seeing the face [69]. Evidence of visual imagery in a cortically blind patient has also been found to involve the fronto-parietal network when imagining an angry person [70]. Indeed, in review of evidence from blindsight studies, Celeghin, de Gelder and Tamietto [71] have suggested that anxiety can be associated with an increased awareness of physiological responses whilst the individual is unaware of the stimuli that precipitated these changes. Furthermore, evidence suggests that individual differences in anxiety influence amygdala response whether there is a conscious awareness of the stimuli or not [5].

It has been suggested that the superior colliculus may have a crucial role in global processing, delivering information directly to extrastriate areas and other subcortical areas areas involved in visual processing, without the need for direct input to V1 [72]. Using evidence from monkeys, Nguyen et al. [73] have suggested that the superior colliculus begins encoding face like stimuli as early as 25 ms post presentation onset. This may indicate an anticipatory monitoring mechanism which precipitates inhibition as the superior colliculus projects to the oculomotor areas involved with generating pro and anti-saccades and has been linked to the preparation of saccade generation [74].

We observed evidence in the high socially anxious group for more errors being generated to upright neutral faces relative to both happy and angry faces (with a non-significant mirroring of this pattern observable for the low anxious group). This pattern in the high socially anxious group can be viewed as indicative of inhibitory difficulties being driven by enhanced indecision relating to the nature of neutral faces in this group. We would argue that it is possible this is being driven by the emotional ambiguity this group perceived in a face devoid of clear emotional content, which subsequently required more socio-evaluative processing in the high social anxious group relative to the other groups of participants.

Vuilleumier [75] suggested that neutral information is more dependent on direct attention than emotional information and increased perceptual load has been linked to reduced processing of neutral distractors [76, 77]. This may have made it more difficult to inhibit neutral faces leading to performance deficits in the antisaccade task. It is also possible that neutral faces had been primed by preceding emotional faces. Indeed, Laptate et al. [69] found that biases to neutral stimuli were influenced by the emotional valence of preceding stimuli. Therefore, it is possible that those with higher social anxiety were primed more by preceding emotional faces because emotion as a social evaluative signal is valued more in this group.

Whilst there remains much to do to further our argument, we conclude that we have provided evidence to support our assertion that a rich socio-cognitive load will enhance realistic performance in static face paradigms designed to elicit group differences in socio cognitive behaviour in the context of social anxiety.

There were some limitations with these initial studies however. The inverted control face employed in experiment 2 was not effective in showing that upright facial expressions had any differential effect generally on saccades relative to the inverted stimuli. However, we argued that the control face may not have acted as anticipated, due to increased socio-cognitive processing which may have given it more social relevance even when inverted. In addition, such effects may have been more pronounced with faces with open mouths rather than closed mouths. It should be noted that such faces were selected in order to enhance ecological validity in a social context. However, we propose that it would be useful for researchers to include a non-face control in future studies. An oval matched to size and skin colour may be one possibility, or a scrambled face.

The lack of group differences despite the opposing patterns on errors rates may also reflect the nature of the sample. It is conceded that there may not be a large enough split between groups in terms of social anxiety scores resulting in effects not being as strong as they may have been with a clinical sample. Another important factor to consider when interpreting the results is the fact that depression and anxiety scores were significantly higher in the high anxious group than the low anxious groups in both studies. Although the concurrence of depression and anxiety is a prevalent feature of social anxiety, this may have some influence on the results. It should be noted however that neither depression nor generalised anxiety scores met clinical cut-off levels, so the influence of these factors may have been relatively small. Nevertheless, they should be considered in such studies.

D'Argembeau, Van der Linden, d'Acremont, & Mayers [78] have discussed the issue of the comorbidity of elevated depression and anxiety scores with social anxiety scores at some length and concurred with Field [67] that ANCOVA is not an appropriate method for controlling a factor that differs between groups. Furthermore, considering the significant comorbidity between social anxiety and depression, e.g. [79, 80] it would be unrealistic and artificial to try to isolate the effect of depression or anxiety and then generalise findings to the behaviour of individuals with social anxiety and co-morbidity.

It is suggested that rather than attempting to control for these, researchers may be wise to instead sample socially anxious participants with varying levels of depression and/or generalised anxiety. This approach would also enable researchers to control the influence of individual differences in tasks such as the anti-saccade task to some extent. However, it should be noted that in the current study there was no significant correlation between either depression or general trait anxiety and error rates for any of the facial expressions. Therefore, there is no evidence to suggest that depression or anxiety influenced the difference in error rates across facial expressions in the high social anxiety group.

Given the apparent effect of the socio-cognitive load in increasing the social salience of the emotional faces, it will be useful for researchers to manipulate different types of cognitive load in a replication of this study in order to assess the relative contribution of social context to the current results. Although there were no differences in latencies or errors as a result of prime types, the additional processing of social information is likely to have increased the social relevance of the faces. However, to be sure that it was the social content that relevantly contributed, a logical next step is to replicate the study with perhaps a mathematical load to measure the effect of processing non-social information on inhibition of faces.

In conclusion, we have argued that conditions of increased social salience are necessary to minimise potential individual differences in performance, and such increases are better engendered through the use of priming techniques than through the use of a potential performance evaluation. Under conditions which do increase social salience, angry faces appear to be indicative of a social threat. This is associated with a general avoidance response indicated by more efficient processing before making an eye movement away from such a face, compared to a more desirable socially affirming face. In addition, there appears to be an exaggerated avoidance response for emotional faces in more socially anxious individuals. However, the ambiguity of a neutral face may be more difficult to inhibit for socially anxious individuals, possibly due to the increased social-evaluative processing required for this task. These findings have implications for static face processing paradigms that lack a personally relevant socio-evaluative component as results may not necessarily reflect the ambiguity elicited by processing the self-relevant context of the face.
